# The four principles of bioethics in cases of Anosognosia

**DOI:** 10.1017/S1092852925100369

**Published:** 2025-07-14

**Authors:** Rennie Burke, Katherine D. Warburton

**Affiliations:** 1 Contra Costa Regional Medical Center, Martinez, CA, USA; 2 California Department of State Hospitals, Sacramento, CA, USA

**Keywords:** Bioethics, Anosognosia, Paternalism, Autonomy, Psychosis, Justice, Beneficence, Nonmaleficence, Psychiatry, Insight

## Abstract

Anosognosia, a term that denotes a lack of insight into one’s own condition, is a defining characteristic of many psychotic illnesses. As a result, generations of psychiatrists have pursued a paternalistic approach to care. Yet in the past century, the overall trend in patient care has been toward autonomy. What does it mean to respect the autonomy of patients whose lack of insight may bring them harm? This chapter will explore these questions through each of the four principles generally employed in bioethical analysis: beneficence, nonmaleficence, justice, and autonomy. Each will have an illustrative case study and explore how anosognosia can further complicate already perplexing ethical scenarios.

## Introduction

When a person rejects treatment for a condition that may literally be killing them due to reasons that are bizarre, irrational, or even delusional, how should we proceed? Psychosis presents a unique bioethical challenge. Anosognosia, the term for a person having impaired insight into the fact that they are ill, frequently afflicts those who suffer from psychotic illness. This lack of insight makes treatment much more ethically complicated. The central issue is the extent to which the doctor weighs the patient’s *autonomy* against their own sense of *paternalism.* Simply put, how do doctors balance the patient’s treatment goals against the doctor’s when there is disagreement? Psychiatrists have embraced both approaches throughout the course of the field’s history, and as we will see, the current absolutist trend toward autonomy may ultimately be causing more harm than good. Before exploring this question in depth, however, some context is necessary. Why is autonomy important to medical ethics, and where did the idea come from?

While there are multiple approaches to bioethics, the one that has achieved primacy in American medical school education, and which therefore permeates the thinking of many healthcare professionals, is principlism.^1^ Principlism, as the name suggests, is the application of a set of principles to help solve moral problems. While these principles vary, the most popular formulation was developed by the bioethicists Tom Beauchamp and James Childress. This model proposes four core principles for medical ethics: autonomy, beneficence, nonmaleficence, and justice. Though there are other models which include principles such as utility, truthfulness, fidelity, and confidentiality, it is Beauchamp and Childress’s formulation that reigns supreme. The challenge of principlism is not so much what principles one chooses as ethical pillars but in applying those principles. Inevitably, principles come into conflict. For the purposes of this chapter, the most widely-embraced bioethical principles of autonomy, beneficence, nonmaleficence, and justice will be examined through several cases to explore the treatment of patients with anosognosia.

## Body

### Case 1: Nonmaleficence

*A 43-year-old man arrives at the emergency room by ambulance looking disheveled and emaciated. He was found lying behind a convenience store dumpster rambling incoherently, wearing 4 sweaters despite the 102* °*F (38* °*C) weather. Routine bloodwork reveals severe anemia as well as several electrolyte abnormalities related to severe dehydration. He can provide his name and knows the date and place, but does not understand the situation he is in. Review of medical records indicates he has a diagnosis of schizophrenia and a long history of taking multiple antipsychotics, most notably the powerful antipsychotic clozapine. He refuses to drink water because it has not been “operated upon.”* Instead, he requests only coffee and energy drinks, which he calls *“life liquids.”*

Nonmaleficence has a long history in medicine, stretching back to Hippocrates in ancient Greece.^2^ In essence, it holds that a doctor should do no harm, which seems straightforward. Harm, or the potential for harm, is an inevitable part of all medical treatment. Every surgery, medication, or even laying on of hands may have a negative consequence for the patient, even if not intended. Nevertheless, if the balance comes out to be on the side of the patient’s well-being, and an effort has been made to minimize the harms, then a physician has abided by the principle. Psychiatric treatment in this respect is no different from other fields. The side effects of antipsychotics—which can include weight gain, movement disorders, and other harms—must be accounted for and tailored to the patient’s unique situation.

Complexity stems from whether doing harm requires actually *doing* something. Can doing *nothing* constitute a harm? In the case above, one could simply treat the patient’s dehydration and electrolyte abnormalities and release him back to the community. Though not *doing* anything actively harmful or cruel to the man, discharging him without treating his underlying condition might be construed as a harm. Indeed, in this scenario, the patient may simply return to the exact same circumstances that led him to the emergency room, only with more lethal results. The Hippocratic exhortation against doing harm, then, may not be as simple as it initially seems. “Not doing” may result in doing harm.

To be sure, there is a dark side to nonmaleficence. For centuries, paternalism was the order of the day in medicine. The Hippocratic age of medicine’s paternalistic practices was closely intertwined with the notion of not doing harm. Doctors routinely withheld information from patients or decided themselves what was the best course of action on the basis of potential harms. You may have entered a surgery intending to lose your appendix, but you could wake up bereft of a gallbladder, too, if a doctor determined that that organ needed to go. If allowing a patient to make a harmful decision or even telling a patient about their condition might cause harm, then the physician could not do it. Paternalism, then, has been embedded in medicine from its very foundations, and its conflict with nonmaleficence is not new.

Many ethical principles, nonmaleficence included, are understood to operate on a sliding scale. If a patient has a cold and declines symptomatic treatment, the doctor can rest easily knowing that not treating those symptoms is unlikely to result in a seriously harmful outcome. In other scenarios, when not doing anything may be the difference between life and death, the application of the principle becomes thornier. As we will see, in the practice of psychiatry, the principle of nonmaleficence frequently comes into conflict with nonmaleficence as well as the other principles. When insight is absent, and physicians uncritically uphold a patient’s right to choose harmful outcomes for themselves, nonmaleficence comes squarely into conflict with autonomy.^3^

### Case 2: Beneficence

*A 33 y/o man presents to the psychiatric emergency room rambling incessantly about the “Alpha and the Omega” and “Judgment Day.” His religious realizations were prompted in part by the voice of God, which he states had spoken directly to him and commanded him to share a new revelation with humanity. He is difficult to interrupt and redirect, though he is able to engage to some degree on religious topics. He appears well-groomed and states he is housed, has a romantic partner, and had a job he recently quit so that he could spread his religious teachings. He states that though he felt stable on his past medications, he wanted to try living without them. After stopping his medications, he experienced his religious epiphanies. Though obviously psychotic and reluctant to take medication, he is open to trying something that will make him “think more clearly.” His decision required a long conversation and a significant amount of persuasion. Chart review indicates that he has a history of schizoaffective disorder, bipolar type and has historically done well on medications, with long periods of lucidity and employment. An antipsychotic that he has done well on in the past is prescribed, and within 2 weeks, he has returned to a nondelusional baseline. He is thankful for having been treated and states that he will be more diligent about taking his medication in the future.*

Beneficence, the idea that a doctor’s actions should have a positive effect on the patient, may also depend on action or inaction. Beneficence can be justified on the grounds of its outcome—does the action maximize the amount of good that can be done for a person? Called *consequentialism*, the idea that an act is moral because its consequences are good has been a way of evaluating the morality of an act since the time of the Ancient Greeks.^4^ This is not the only way to understand beneficence, however: it can also be based on the intrinsic value of doing good regardless of the outcome. This concept of morality, equally ancient, is embodied in the law codes both holy and secular that bar murder or exhort truth-telling irrespective of the consequences. Whether by divine mandate or by Immanuel Kant’s “Categorial Imperative” for rational subjects to act only according to principles they would want to be universal, *deontology* holds equal influence in the world of bioethics.^5^

Regardless of whether it is based on the outcome or the intrinsic nature of doing good, beneficence is a cornerstone principle of modern medical practice. In the case above, a patient with a history of living independently in the community while taking psychiatric medications arrived at the hospital in a highly decompensated state after he stopped taking them. Though he is highly psychotic and seems to lack insight into the relationship between his medications and his delusional and religiously preoccupied state, he is still able to consent to taking medications which restore him to a state of insight. The most obvious act of beneficence in this case would be to provide him with these medications.

What amount of persuasion is appropriate, and at what point should we allow him to persist in his delusional and hallucinatory state, knowing that he has a track record of doing well taking medication? Paternalism again rears its head depending on how one views the nature of the persuasion that occurred.^6^ As with the case of nonmaleficence above, the principle of autonomy may clash with beneficence. The extent of the clash depends on how much one chooses to accept “no” for an answer to the question of taking medication. When exactly a doctor’s efforts at persuasion become inappropriate is a complex question. Again, the appropriateness of this persuasion is a sliding scale: the more consequential the decision, the more avid the persuasion is likely to be. Though psychotic illnesses may very well be akin to dementia as neurodegenerative, American culture’s respect for autonomy entails treating the initial “no” to medication as the final word, no matter how psychotically motivated.^7^ This standard of care, which eschews assessment of capacity for imminent danger to one’s self or others, is the subject of our next discussion.

### Case 3: Justice

*A disheveled woman arrives at the psychiatric emergency room after climbing onto a golf course and attacking an empty golf cart. She has been seen roaming the woods behind the golf course before, at times sleeping there, and has also been observed eating out of the trash can behind the snack bar. Though the weather is quite cold, she is wearing light, thin clothing. Her thinking is highly disorganized, and she arrives by ambulance screaming and refusing to participate in any staff attempt to interact with her. In her private room at the emergency department, she is demanding to leave and return “home,” but she cannot provide an address. She is adamant that there is nothing wrong with her. Her worried family arrives at the ED and describes increasingly bizarre behavior, which now includes wandering from the house at night and not coming home. After a workup, she is given a diagnosis of neurodegenerative disease: Alzheimer’s. A social worker meets with the family to help connect them to the multiple resources available through the Alzheimer’s Association and local non-profits. They are given a pamphlet describing dementia care practice recommendations, which outlines the comprehensive, person-centered biopsychosocial standard of care for this condition. The compassionate staff members give the family a list of locked memory care facilities and recommend placement in a locked environment if she continues to wander. They describe the typical medication algorithms that are recommended to slow the progression of her disease and help her overcome her agitated mood. Due to her anosognosia, she will now have a family member help make the decisions about when she will go to a memory care center, perhaps locked, and authorize doctors to start a medication to help preserve her memory.*

Imagine if this patient’s diagnosis was schizophrenia, a condition now also understood to be a neurodegenerative disorder. In that case, she would be allowed to leave the emergency department with no treatment or resources. Her family would be told that there is nothing the hospital staff can do to help her because she does not want treatment and is not dangerous to herself or other people. They will be told that sleeping in thin clothes in the woods behind the golf course during inclement weather does not rise to the level of dangerousness needed for them to intervene. Neither will her behavior of eating out of trash cans at the golf course. Even if these behaviors cause her to be arrested and incarcerated, the hospital staff would still not be able to intervene. Why does our society treat these two neurodegenerative disorders so differently? Is this justice?

The ethical principle of justice concerns fairness, and this is especially salient in discussions about the way we have distributed our resources to treat mental illness.^8^ The financial implications of this distribution will be treated elsewhere in this book. For our purposes, justice also includes how we allocate other, equally precious resources: our attention and compassion. A tragically common feature of life in American cities is a disheveled person with a psychotic illness talking to themselves in the street as passersby ignore them. That this person is clearly suffering makes no difference in how much care the world at large chooses to give them. This same attitude can permeate healthcare interactions, too. Our attention and who we give it to are tragically limited.

The explanation for this striking disparity may lie in a contrast with the treatment of that other large group of patients with anosognosia, those with dementia. These patients are often treated with dignity and understanding. Patients with schizophrenia, by contrast, are often denied the same dignified treatment because of perceptions about the alienness of their thinking, talking, and acting. Providers are likely not completely conscious of the inequitable distribution of their care because a psychotic patient’s protests may not even register—some patients who have prominent “negative symptoms” of social withdrawal and disorganized thought can only register their discontent in the most indirect ways. Moreover, their distorted relationship with reality and the behaviors it can lead to at times provoke strong feelings of fear and even dislike from providers. Yet if we hold up justice as a value worth striving for, this inequitable distribution of care should be unacceptable.

There is also a striking disparity in the way we evaluate the ability to make decisions by patients who lack insight. Patients with dementia routinely undergo evaluations of whether or not they can make decisions about their health, and there are widely followed conventions and regulations about how to involve them in this process.^9^ The traditional criteria for evaluating capacity, set forth by ethicist Paul Appelbaum, consists of the ability to understand information, to appreciate the consequences of acting or not acting, to reason about the information provided, and the ability to communicate a decision.^10^ If a patient lacks one of these abilities, then they are said to lack the capacity to make a specific medical decision. Capacity can wax and wane as people recover or lose their faculties, as in delirium.^11^ In some instances, when there is a global decline in capacity—as with dementia—a person can be found to lack competence and have all medical decision-making taken away. In our case above, the patient lacks capacity to make treatment decisions, if not competence entirely. Her cognition, as well as her insight into her condition, is impaired by illness, as judged by a medical professional.

With mental illness, a different standard is routinely employed: that of dangerousness. If a patient is behaving in a way that may endanger themselves or others due to their mental illness, then they may have their ability to make their own medical decisions stripped from them. In practice, this is a much higher standard. Even in instances when their presenting condition may be driven by an underlying psychotic—and indeed, neurodegenerative process—they are allowed to make their own decisions so long as they are not overtly “dangerous.”^12^ With our patient above, eating out of the trash and living lightly clothed in cold outdoor weather were not dangerous enough to rise to the level of a legal hold. The reasoning often goes that there are people with no illness who pursue this lifestyle out of choice, so why should doctors be able to deprive patients of that life choice? In many instances, the patient will continue to deteriorate until there is a life- or limb-threatening condition, at which point they will be placed on a legal hold. In many instances, it is too little, too late. Is this allocation of our mental energies toward capacity assessments for dementia patients and dangerousness assessments of the mentally ill just? Or is there an asymmetry between these two similar groups of patients that has gone unexamined?

Whether it is financial resources, time, or our hearts, there is a clear miscarriage of justice with respect to the care of people with psychotic illness. Unlike neurodegenerative dementia disorders, whose progress can only be slowed and not reversed, patients with schizophrenia can be restored to levels of function that had previously been lost. Currently, individuals with severe psychotic illnesses are cycling through forensic hospitals, carceral settings and the open streets, when they could be, with proper treatment, living in the community and flourishing. The present situation violates the ethical principle of justice on an individual and a systemic level, but there are ways forward. Models of care developed in other countries show that people with severe psychotic illness and low insight can live in the community and lead meaningful, rich lives.

### Case 4: Autonomy

*A 27-year-old man arrives at a hospital in the custody of a local official, complaining that there is a gang of people controlling his thoughts and behaviors with an “air loom,” a gigantic weaving device whose invisible threads have become attached to his body. The “air loom gang” did not just attack him, however: they use their contraption’s gases and rays to control politicians around the world and are inspiring wars and revolutions. This conviction led him to disrupt the proceedings of the national legislature in spectacular fashion. His speech is replete with odd phrasing and neologisms, and he is tormented by his paranoia. His only seeming wish is that the operators of the air loom be tracked down and brought to justice. He declines psychiatric help and insists that the real problem is the existence and activities of the “air loom gang.”*

James Tilly Matthews, the subject of this case study, was first admitted to the hospital on January 28th, 1797. A record of his delusions and treatment comprises what may be the first case study in schizophrenia ever published, first appearing in London in 1810.^13^ Matthews lived much of the remainder of his life in Bethlem Royal Hospital in London, England. Though relatively harmless, he was perceived to be a “maniac,” and committed over the objections of his family, who fought through the legal system to keep him free and living with them. Doctors of the time felt that to treat him required involuntary commitment. Ultimately, after significant agitation by his family, he was transferred to a private asylum in 1814 and died one year later, in 1815.^14^

Matthews’s experience encapsulates the debate about how much autonomy should be accorded to patients with anosognosia, a debate which continues to this day. Would Matthews meet the criteria for hospitalization now? According to a strict legal definition, he most likely would not: he was eating, clothing himself, and living independently (and thus not “gravely disabled”), though in the community he was causing disruptions to the British parliament and experiencing extreme, psychotic paranoia. Nevertheless, there is an attitude among some that patients such as Matthews can only improve if they are committed to an inpatient unit, possibly indefinitely.

To place a patient such as Matthews on an inpatient unit indefinitely would represent, then as now, a failure of care. First, it would be unjustified. Unlike the other cases above, Matthews was not gravely disabled, nor was he a threat to his own life or the lives of others despite his erratic behavior. Proactive treatment in the community and connections to resources that would allow him to continue independent living would be ideal. This was the promise of 20th-century legislation regarding mental health and in part justified the drive for deinstitutionalization that resulted in state hospitals being closed. Unfortunately, the promised community treatment never materialized. Patients like Matthews suffered, as did patients such as those in the cases that preceded the one above.

As the case of Matthews shows, anosognosia has been evident in society in some recognizable form for hundreds of years, and likely much longer. It is in this context, in which patients might not be able to clearly articulate their wishes, that the appeal of paternalism is strongest. Indeed, it *has been* strong. Psychiatry, it should be acknowledged, has had an ugly history of paternalism. Asylums born of a progressive, reforming instinct rapidly became overcrowded and the site of forced procedures to “cure” mental illness. From insulin shock therapy (in which patients were put into hypoglycemic comas) to the notorious lobotomy, (which shredded the neural connections in the prefrontal cortex) these treatments were generally ineffective and often barbaric. Yet this was not the perspective of mainstream medicine: the lobotomy’s inventor, Antonío Egas Moniz, won the Nobel Prize in Medicine for its discovery.^15^ In one instance, a psychiatrist in New Jersey’s state hospital system took the notion of psychosurgery to such an extreme that he began removing tonsils, gallbladders, appendices, teeth, and ultimately sections of the colon to cure mental illness.^16^ The historical record of people who have suffered at the hands of paternalistic psychiatrists who saw themselves as benevolent is indeed long.

A number of factors transformed the paternalistic approach over the course of the 20th century. The Nuremburg Trials of Nazi war criminals revealed the routine, barbaric violation of the autonomy of concentration camp inmates in the name of “research,” though the experiments themselves were often mere pretexts for cruelty. These violations led to the promulgation of an ethical code for medical research, the Nuremburg Code, which enshrined patient autonomy as its first principle.^17^ Though an extreme example, this laid bare the coercion that was inherent in many contemporary medical practices, including the much more mundane. The medical world was rocked again when Dr. Henry Beecher released a landmark paper outlining violations of patient rights in various medical experiments in the years after Nuremburg. Comfortable in their conviction that they were far removed from the Nazis in time, geography, and behavior, the medical establishment continued to routinely violate patient autonomy and indulge its worst paternalistic impulses.^18^ The current standards for medical research ethics, 1964’s Helsinki Declaration, and 1979’s Belmont Report, are routinely updated in light of changing technology—and continued violations of medical and research ethics.^19^^,^^20^

Given the horrors that were visited on many people with mental illness, the arc of development of the last 75 years has been toward restoring autonomy to psychiatric patients. While some of this was spurred on by a growing antipsychiatry movement, supporters came from all corners of the political spectrum. This advocacy resulted in legislation such as the federal Community Mental Health Act and bills like California’s Lanterman-Petris-Short Act.^21^^,^^22^ Psychiatric patients who had once been institutionalized found themselves able to exercise autonomy over their lives and health. Yet as we explored above, the effects of this have often been negative for patients themselves. As their neurodegenerative disease progresses untreated, their situations can become increasingly desperate, and many find themselves in significant danger that they lack the insight to recognize or abate. Has the pendulum swung too far in the direction of autonomy?

Balancing autonomy and paternalism is especially vexing in psychiatry. As in the cases above, individuals with psychotic disorders do not always recognize the extent to which symptoms impact their lives, or even whether they have a disease at all. This need not be inconsistent with autonomy; regardless of how psychotic a patient may be, they may still maintain their core perspectives on life and death and be able to articulate them fluently. Yet when it comes to whether or not they should be taking medication, enter the hospital, or even have the ability to make decisions about where they live, mental health professionals and patients with anosognosia may find themselves at odds.

In protecting patients from their lack of insight, we must be careful to respect their autonomy and maintain a high standard for treatment against their will. In our case series, we saw people whose insight was impaired to the point that it made it impossible for them to live successfully in the community with our present resources. But we also saw Matthews, who similarly lacked insight but had survived without issue with his family’s support prior to his lengthy institutionalization in Bethlem Hospital.^22^ As it is with people who have insight, we must have a high standard for overriding a patient’s autonomy and abandoning hope that they could live in the community. Matthews did not seem to meet this criterion, but the norms of the time mandated that someone as paranoid and psychotic as he was be institutionalized. We must be careful not to make the same mistakes.

As noted above, 20th-century legal developments concerning severe psychosis have been in the direction of greater autonomy. Patients who had formerly dwelt in state hospitals were released to the community in part because this was thought to be less of a constraint on their autonomy. This could have been a positive development. Exposés about the nature of asylums are numerous, and the inhumane treatment and overcrowding necessitated reform if not revolution. But current outcomes for people living with schizophrenia suggest that there may have been an overcorrection. Patients like the above are routinely in danger or endangering others. Their seemingly autonomous decisions are not actually autonomous, but rather weighed down by decades of mental illness, often untreated. Respecting their desire to not drink water or to eat trash behind a golf course on the grounds of respecting their autonomy does a disservice to them and their community. There are a range of options currently available, from intensive mandated or voluntary outpatient treatment to inpatient hospitalization of varying durations (including indefinitely), but all the solutions have one thing in common: paternalism. The echoes from the medical past therefore remain with us. What is needed is a way forward that both respects a patient’s autonomy but also gives them the best chance to utilize their autonomy in the community in all the ways that make life meaningful. Community-based care, models for which exist elsewhere in the world, and which will be described in other chapters, serve as examples that could one day influence how care is delivered in the United States.

## Conclusion

Finding a balance between the push and pull of autonomy and paternalism for patients with anosognosia will not be easy. The answer may lie in incorporating other principles outside of Beauchamp and Childress’s original four, as well as more observable changes in a patient’s well-being and our own inner feelings. All of these factors could resolve some of the tensions that existing principles have with one another in the case of psychosis with anosognosia.

We explored in the paragraphs above how patient autonomy often runs afoul of the paternalistic impulses of doctors who want to treat a problem even if it’s against a patient’s wishes. In a different vein, beneficence and nonmaleficence come into conflict with justice when resources are limited, and one cannot do an equal amount of good for all parties involved. And most notably, as it is the basis of some of the cases above, beneficence and nomaleficence conflict with autonomy when respecting the desires of a patient with anosognosia lead to conditions which may result in severe harm or even death to a patient. In all these cases, the four chief principles of bioethics are in tension. What can we do to resolve this conundrum?

One answer may be to consider other principles in conjunction with our four above. Transparency and truth-telling are two principles that could be helpful. Through a commitment to transparency, discussions around voluntary or even compelled treatment would become more collaborative as the decision-making is laid bare to the patient and the scope of who is involved in care is dramatically expanded. The approach of open dialogue promises just this. In open dialogue, the approach of dialogic practice entails creating a broad, interdisciplinary and intercommunity team which includes a patient’s friends and family.^23^ This group discusses a patient’s situation with the patient in an effort to foster an appreciation for multiple perspectives, not just those of the clinicians. This even includes discussions of things the patient may lack insight into, such as hallucinations or delusional systems. Transparently sharing perspectives and experiences between the two parties respects the patient’s autonomy regardless of their level of insight.

More concretely and less in the realm of principles, improving quality of life in concrete ways may also inform our conceptions of beneficence, justice, autonomy, and nonmaleficence. In the United States, over untold thousands of people living unhoused experience mental illness. An important first step, then, could be working to provide housing for this population as a prerequisite to treatment. The stability that housing affords could immeasurably improve the quality of life. So too could employment, as patients with severe mental illness report enhanced quality of life in one analysis.^24^ Though these may also be proxies for other factors such as social connectedness, enhancing the material conditions of patients in and of itself could be a kind of principle to strive for.

Over and above the principles we have explored, a robust sense of compassion would do well in serving the principles of justice and beneficence. As we alluded to above, there is a disparity in the distribution of emotional energy in the treatment of patients with psychotic patients who have anosognosia. A compassion that derives not from a condescending sense of noblesse oblige but instead relating to a patient’s experiences, however different, would enhance the quality of care delivered and go a long way toward addressing the asymmetries of care in treatment. Compassion is a force amplifier that enhances the positive effects of treatment and aligns well with the principles that undergird medical treatment.

Ultimately, guaranteeing better treatment for patients with anosognosia will require a broader array of resources than we currently employ. Yet, this need not be *more* resources. The United States spends significant amounts of money incarcerating patients with anosognosia in prisons and jails, remanding them to indefinite stays in inpatient facilities and hospitals, and attempting to provision numerous services to them in non-ideal settings such as the street. Yet around the world, there are numerous people with serious mental illness and anosognosia who live successfully in the community. Models such as that in Trieste, Italy show that this is not simply a speculative dream, but a reality that can be emulated. It will be up to us to build a brighter future.
